# Supplementary material for: Associations of Serum Retinol and α-Tocopherol Levels with Uric Acid Concentrations: Analysis of a Population-Based, Nationally Representative Sample
Source: Nutrients. 2020 Jun 17;12(6):1797. doi: 10.3390/nu12061797 (PMC7353330; doi:10.3390/nu12061797)
Supplement: Supplementary file 1 [file nutrients-12-01797-s001.pdf]

Supplementary Table S1. Association of vitamin A intake with uric acid levels.

	Q1	Q2	Q3	Q4	Q5	P trend*
Total						
Retinol intake						
Model 1*	5.23(5.14,5.32)	5.09(5.01,5.17)	5.17(5.09,5.25)	5.14(5.06,5.22)	5.20(5.12,5.28)	0.858
Model 2†	5.22(5.13,5.30)	5.12(5.04,5.19)	5.16(5.08,5.23)	5.14(5.06,5.21)	5.19(5.13,5.26)	0.983
Model 3‡	5.23(5.14,5.33)	5.14(5.05,5.23)	5.18(5.08,5.27)	5.15(5.06,5.25)	5.22(5.13,5.30)	0.966
Model 4§	5.24(5.14,5.33)	5.14(5.05,5.23)	5.18(5.08,5.27)	5.15(5.06,5.25)	5.22(5.13,5.30)	0.989
Men						
Retinol intake						
Model 1*	6.01(5.78,6.14)	5.82(5.69,5.95)	6.00(5.86,6.15)	5.87(5.73,6.01)	5.99(5.86,6.12)	0.821
Model 2†	6.05(5.93,6.18)	5.85(5.73,5.98)	5.97(5.83,6.10)	5.85(5.73,5.98)	5.97(5.85,6.08)	0.540
Model 3‡	6.08(5.94,6.22)	5.88(5.73,6.02)	5.99(5.83,6.15)	5.88(5.74,6.02)	6.00(5.86,6.14)	0.610
Model 4§	6.08(5.94,6.22)	5.88(5.73,6.03)	5.99(5.83,6.15)	5.88(5.74,6.03)	6.00(5.86,6.15)	0.608
Women						
Retinol intake						
Model 1*	4.43(4.32,4.55)	4.35(4.26,4.43)	4.39(4.30,4.49)	4.42(4.34,4.51)	4.38(4.29,4.46)	0.849
Model 2†	4.39(4.29,4.49)	4.37(4.29,4.45)	4.38(4.29,4.47)	4.43(4.35,4.51)	4.41(4.33,4.48)	0.480
Model 3‡	4.49(4.37,4.62)	4.48(4.37,4.59)	4.49(4.37,4.61)	4.53(4.40,4.65)	4.50(4.38,4.61)	0.680
Model 4§	4.49(4.37,4.62)	4.48(4.37,4.59)	4.49(4.37,4.61)	4.53(4.40,4.65)	4.50(4.38,4.61)	0.716

Data were presented with mean (standard error) * P for trend were calculated using linear regression analysis by considering quintile distribution of serum retinol and α-tocopherol levels as continuous variables † Model 1 adjusted for age; ‡ Model 2 additionally adjusted for BMI, and GFR; § Model 3 additionally adjusted for residence, education, smoking status, alcohol consumption, physical activity, systolic and diastolic blood pressure, and log transformed hs-CRP; Model 4 additionally adjusted for serum retinol and α-tocopherol, levels mutually.
